# Analysis of factors associated with traffic injury severity on rural roads in Iran

**DOI:** 10.5249/jivr.v4i1.67

**Published:** 2012-01

**Authors:** Ali Tavakoli Kashani, Afshin Shariat-Mohaymany, Andishe Ranjbari

**Affiliations:** ^*a*^School of Civil Engineering, Iran University of Science and Technology, Narmak, Tehran, Iran.

## Abstract

**Background:**

Iran is a country with one of the highest rates of traffic crash fatality and injury, and seventy percent of these fatalities happen on rural roads. The objective of this study is to identify the significant factors influencing injury severity among drivers involved in crashes on two kinds of major rural roads in Iran: two-lane, two-way roads and freeways.

**Methods:**

According to the dataset, 213569 drivers were involved in rural road crashes in Iran, over the 3 years from 2006 to 2008. The Classification And Regression Tree method (CART) was applied for 13 independent variables, and one target variable of injury severity with 3 classes of no-injury, injury and fatality. Some of the independent variables were cause of crash, collision type, weather conditions, road surface conditions, driver's age and gender and seat belt usage. The CART model was trained by 70% of these data, and tested with the rest.

**Results:**

It was indicated that seat belt use is the most important safety factor for two-lane, two-way rural roads, but on freeways, the importance of this variable is less. Cause of crash, also turned out to be the next most important variable. The results showed that for two-lane, two-way rural roads, "improper overtaking" and "speeding", and for rural freeways, "inattention to traffic ahead", "vehicle defect", and "movement of pedestrians, livestock and unauthorized vehicles on freeways" are the most serious causes of increasing injury severity.

**Conclusions:**

The analysis results revealed seat belt use, cause of crash and collision type as the most important variables influencing the injury severity of traffic crashes. To deal with these problems, intensifying police enforcement by means of mobile patrol vehicles, constructing overtaking lanes where necessary, and prohibiting the crossing of pedestrians and livestock and the driving of unauthorized vehicles on freeways are necessary. Moreover, creating a rumble strip on the two edges of roads, and paying attention to the design consistency of roads can be a helpful factor in order to prevent events such as "overturning" and improve the overall safety of freeways.

## Introduction

Iran is a country with a high rate of road traffic crash fatality and injury. According to statistics from the Forensic Medicine Organization of Iran, between 2006 to 2008, traffic crashes resulted in an average of 24 000 people (i.e. 3 persons per hour) dead and around 240,000 cases injured, annually.^1^ A considerable amount of research has been carried out in order to understand the circumstances under which drivers and passengers are more likely to be killed or more severely injured in an automobile crash, and to recognize the factors affecting the severity of crash-related injuries. Thus by preventing crashes and also by reducing their severity, the overall driving safety situation may be improved. Chang and Wang^2^used 2001 crash data for Taipei, to establish the relationship between injury severity and driver/vehicle characteristics, highway/environmental variables and crash variables, and concluded that the most important variable associated with crash severity is the vehicle type. Yan and Radwan^3^performed an analysis of the relation between rear-end crashes occurring at signalized intersections and a series of potential traffic risk factors. Analyzing the 2001 Florida crash database, they found that rear-end crashes are over-represented in the higher speed limits (45–55 mph), and the danger of a rear-end crash is greater during daytime, in wet and slippery road surface conditions, with male drivers, and drivers younger than 21 years old. Another study related to the Ethiopian capital, Addis Ababa, showed that speeding and neglecting pedestrians’ right of way were the most significant causes increasing injury severity.^4^

More than 90 percent of passengers in Iran travel by road,^5^and rural roads play a significant role in this transportation. In addition, in Iran 70 percent of the fatalities happen on rural roads.^1^So the need to conduct a concerned study is undeniable. The main objective of this study is to identify significant factors influencing injury severity among drivers involved in crashes on rural roads in Iran.

The study was performed on all the crash data, pertaining to a 3-year period (2006-2008), on two major types of rural roads: two-lane, two-way roads and freeways. Analyzing two different patterns of roads with such geographical vastness and large amount of data is almost unknown among all the previous studies.

## Methods

In this study, the Classification And Regression Tree (CART) method has been employed to classify the target variable of injury severity and find significant factors influencing injury severity among drivers involved in crashes on rural roads of Iran. Further explanation of the data and analysis method is given below.

**Study Area**

The 21 579 km of two-lane, two-way rural roads and 1 606 km of rural freeways comprise a significant proportion of the rural road network of Iran. Since traffic patterns on these two types of roads are different, they can account for a good sample of all rural roads of the country. 

**Data Treatment**

The primary source of the crash data required to perform this study was statistics from the Information and Technology Department of the Iranian Traffic Police; from all the crash data on the police database related to different types of roads, we extracted all the crash data related to two-lane, two-way rural roads and rural freeways. The dataset is created from a total of 213 569 drivers involved in crashes that took place on rural roads in Iran, over the 3-year period. 169 648 of these drivers were driving on two-lane, two-way rural roads, and 43 921 of them on rural freeways. These data are obtained from the Iranian traffic crash record form, known as KAM 114, which contains important information about the crashes. In Iran, the police officer at the crash scene fills in the different parts of the crash form. And since being at-fault or the existence of some special causes for the crash, carries strict penalties for drivers, the police officers are trained for this task and are asked to carry it out properly and accurately.

Table 1 presents the aforesaid information in terms of 13 independent variables, and one target variable of injury severity with 3 levels of no-injury, injury and fatality. It should be noted that since the study concerns motor vehicles, we eliminated cases related to motorcycles, bicycles and vehicle-pedestrian collisions.

**Table:** Table 1:**Variable description**

Description	Variable
Target variable: 1. No-injury 2. Injury 3. Fatality	Injury severity
1. Male 2. Female	Gender
Continuous	Age
1. Used 2. Not used 3. Unknown	Seat belt
1. Following too closely 2. Ignoring proper lateral distance 3. Ignoring right of way 4. Inattention to traffic ahead 5. Lack of driving skill 6. Lack of vehicle control 7. Speeding 9. Improper overtaking 11. Straying to the right 13. Illegal turning 14. Crossing at prohibited place 15. Driving on the wrong side of the road 16. Improper backing 17. Vehicle defect 19. Swerving 20. Pedestrian violation 21. movement of pedestrians, livestock and unauthorized vehicles on freeways 22. Improper packing 23. Improper towing 24. Red light running 25. Turning in no-turn zone 26. Other	Cause of crash*
1. Collision with motorcycle/bicycle 2. Two vehicle collision 3. Multi vehicle collision 4. Collision with pedestrian 5. Collision with animal 6. Fixed object collision 7. Overturning 8. Fire/Explosion 11. Other	Collision Type**
1. Auto 2. Mini bus 3. Bus 4. Pickup 5. Light truck 6. Truck 7. Ambulance 8. Truck with trailer 11. Agricultural vehicles 12. Highway const. equipment 13.Fire truck 14. Police car 15. Other	Vehicle Type***
1. Segment 2.Intersection 3. Bridge 4. Tunnel 5. Roundabout 6. Other	Location Type
1. Daylight 2. Dark 3. Dusk/Dawn	Lighting Condi-tion
1. Clear 2. Fog 3. Rain 4. Snow 5. Stormy 6. Cloudy 7. Dusty	Weather Con-dition
1. Dry 2. Wet 3. Icy 4. Gravel/Sand 5. Slush/Mud 6. Oil spill 7. Other	Road Surface Condition
1. On roadway 2. On Shoulder 3. In median 4. On roadside 5. Outside traffic way 6. Other	Occurrence
1. None 2. Stabilized gravel 3. Paved	Shoulder Type
Continuous	Shoulder Width

* Cases No. 8, 10, 12 and 18 are not in the dataset. ** Cases No. 9 and 10 were related to motorcycles and pedestrian collision. ***Cases No. 9 and 10 were motorcycles and bicycles.

According to Breiman,^6^ in order to develop a CART model, the dataset should be randomly divided into 2 subsets of training and testing. In this study, the model was trained by 70% of the data, and tested with the remaining 30%. The analysis was performed on two major types of rural roads; therefore, two CART models were developed, one for rural two-lane, two-way roads and another one for rural freeways. The predictors and target variables are the same for both models; however the data for each model was analyzed separately.

**Classification And Regression Tree method (CART)**

Data mining is the discovery and analysis of a large amount of data to find meaningful models and patterns.^7^Considering the large amount of data available on crashes which take place on rural roads in Iran, data mining was found to be a suitable approach for this study. One of the most important and popular data mining tools, which has been widely employed in different fields of research is the Classification And Regression Tree (CART). With no pre-defined underlying relationship between the dependent and independent variables, CART turned out to be a powerful method for dealing with prediction and classification problems, particularly when there is a large amount of data with many independent variables.

Classification is a process of analyzing and developing models in order to describe and define the important classes, and predict their future behavior. The principle of the CART method in developing the decision tree is such that, at first all data are concentrated at the root node, located at the top of the tree. Then, it will be divided into two “child” nodes, on the basis of an independent variable, which creates the best homogeneity. In fact, the data in each “child” node are more homogenous than those in the upper "parent" node. This process will be continued repeatedly for each “child” node, until all the data in each node has the most possible homogeneity. Such a node is called a terminal node, and has no branches.

One of the most commonly applied indexes for splitting the classification tree is the Gini index, shown as follows:

**Figure:**
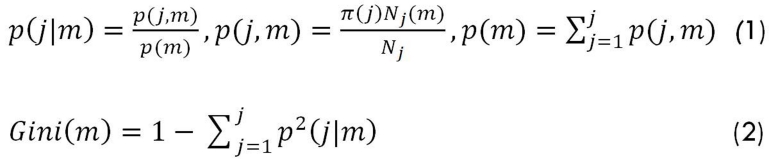


where J is the number of classes or the target variables, π(j) is the prior probability for class j, p(j|m) is the probability that node m includes observations of class j, and Gini(m) is the Gini index ,which indicates impurity in node m.^8^The Gini index is equal to 0 when all the observations in one node belongs to a unique class, which shows the least impurity, and is equal to 1-1/j, when there are observations of different classes with the same proportion in one node.

"Misclassification cost" indicates those data which are misclassified. It is computed through the below equation, and can be utilized as a goodness of fit measure:

Misclassification cost=

**Figure:**
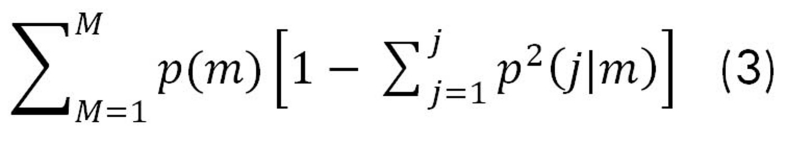


where p(m) is the proportion of observations in the terminal node m to the total observations, and M is the number of terminal nodes.^8^

In the CART method, the decision tree grows more and more until there are the same observations in each terminal node. In this situation, the maximum tree has been generated which overfits the training data. To reduce the complexity of the final tree and generate simpler trees, the tree will be "pruned" on the basis of a cost-complexity algorithm. The simpler a tree is, the higher is the misclassification cost. Therefore, after cutting off a sub-tree, if the increase in misclassification cost is sufficiently less than the complexity rate reduction, the branch in question will be trimmed and a new tree is generated. The optimal tree will be selected from the pruned trees, such that: with an increase in complexity (more terminal nodes), the misclassification cost repeatedly decreases for training data, whereas for testing data, first there is a decrease and then an increase. An optimal tree is the one which has the least misclassification cost for testing data.

Another significant output of the CART method is the "Variable Importance Measure" (VIM), which can be utilized for variable selection procedure. In a classification tree with T total nodes, let S(Xj, k) be the split at the kth internal node using the variable Xj. The VIM for this variable is the weighted average of the reduction in the Gini impurity measure, achieved by all splits using the variable Xj across all internal nodes of the tree and the weight is the node size. If N is the total number of observations in the training sample, then the formula for the importance for variable Xj is given by the following:

**Figure:**
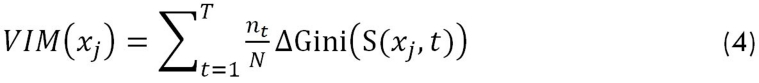


where ΔGini(S(Xj,t))represents the reduction of Gini index on the basis of variable Xj in node t, and nt/N represents the proportion of the observations in the dataset that belongs to node t.^9^

In this study, however, VIM values are computed for each of the independent variables individually and then the values are scaled in such a way that the sum of all variables' VIM will be equal to 1. The variable that has the most importance compared to others gets the relatively greatest number, as well.

## Results

The CART model that was employed to predict the target variable, had 13 input variables and a single output one with 3 classes of no-injury, injury and fatality.

**Fig. 1 Decision tree of the freeways model:**
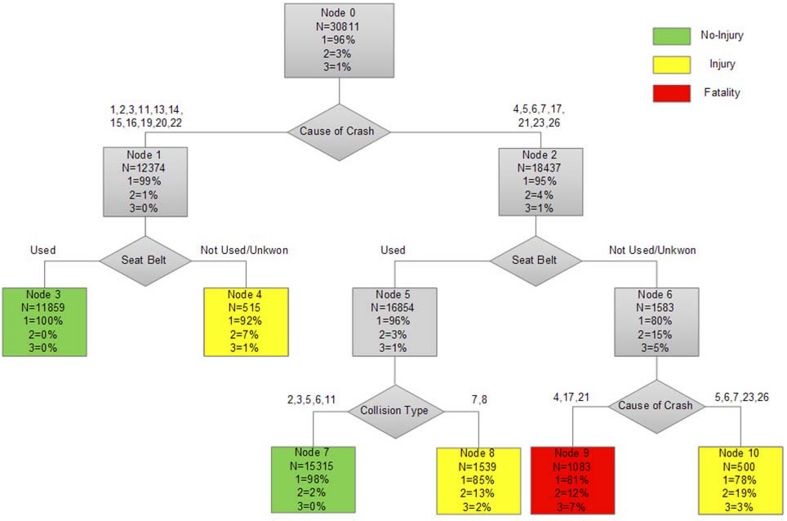


One of the advantages of the decision tree over the other modeling methods is that it helps the decision makers to answer the "if-then" questions easily. In addition to this, the CART model can be easily understood and interpreted because of the graphic nature of its results. To give an example, we have shown the decision tree diagram for the freeways model in Fig.1, and the interpretation of the results is discussed in the following section. But before that, it is important to note that, due to the similar importance of the aforesaid 3 injury severity classes, prior probability π(j)was set equally for them:π(j)=0.33.So, in this tree analysis the classifications are a bit different from other studies. That's why some node boxes (Nodes 4,8,9 and 10) are classified under a class label, which does not necessarily have the highest percentage.

Applying the classification method, the relative variable importance of all the 13 independent variables is also computed for both models of two-lane, two-way rural roads and rural freeways. The corresponding results are presented in Table 2.

**Table:** Table 2:** Relative importance of variables**

VIM for freeways	VIM for two-lane two-way roads	independent variable
0.2137	0.8214	Seat belt
0.3773	0.1484	Cause of crash
0.2858	0.0088	Collision type
0.0197	0.0029	Vehicle type
0.0115	0.0023	Weather conditions
0.0115	0.0023	Age
0.0115	0.0023	Shoulder type
0.0115	0.0023	Shoulder width
0.0115	0.0023	Road surface condition
0.0115	0.0023	Lighting condition
0.0115	0.0023	Location type
0.0115	0.0023	Occurrence
0.0115	0.0001	Gender
1.0000	1.0000	Sum

Furthermore, Table 3 represents the prediction accuracies for training and testing data in both models.

**Table:** Table 3:** Prediction accuracy of the models for the three classes**

Testing data		Training data			
Correctly predicted	Observed severity	Correctly predicted	Observed severity		
35736(75.93%)	47063	83463(76.11%)	109656	No-injury	
941(27.90%)	3373	2324(29.93%)	7765	Injury	Two-lane two-way
244(49.39%)	494	665(51.27%)	1297	Fatality	roads
36921(72.49%)	50930	86452(72.82%)	118718	Overall	
10300(81.22%)	12681	24258(81.38%)	29808	No-injury	
114(33.73%)	338	322(40.05%)	804	Injury	
32(35.16%)	91	101(50.75%)	199	Fatality	Freeways
10446(79.68%)	13110	24681(80.10%)	30811	Overall	

## Discussion

As provided in Table 2, for both types of roads, seat belt use, "cause of crash" and "collision type" turned out to be the three most important variables. However, the order and the percentages are different for two-lane, two-way roads and freeways. In the two-lane, two-way roads analysis, seat belt use turned out to be the most important variable, such that it is about 6 times more important than the second variable, and 90 times more than the third one. It indicates that on such roads, there is more probability for a driver who is not using a seat belt to get more severe injury when involved in an accident. This result has been also pointed out in some previous studies,^10-14^and shows the necessity for mandatory seat belt use. But in the freeways analysis, this variable ranks third, which is probably due to strict police enforcement and control of seat belt use on such roads. The other important variable, which ranks first for freeways and second for two-lane, two-way roads, is "cause of crash". This confirms the study of Al-Ghamdi^15^in Saudi Arabia, where cause of crash was recognized as an important factor in increasing crash severity.

The variable importance of the other 10 variables is very low, and they do not play a significant role in predicting the target variable.

As mentioned before, the decision tree diagram for the freeways model is shown in fig 1 as an example. The classification tree firstly segments the data into two groups, based on the variable of "cause of crash": crashes due to causes following too closely, ignoring proper lateral distance, ignoring right of way, straying to the right, illegal turning, crossing prohibited place, driving on the wrong side of the road, improper backing, swerving, pedestrian violation and improper packing (No. 1,2,3,11,13,14,15,16,19,20 and 22) go to Node 1, and crashes due to causes inattention to traffic ahead, lack of driving skill, failure to control vehicle, speeding, vehicle defect, movement of pedestrians, livestock and unauthorized vehicles on freeways, illegal towing and other (No. 4,5,6,7,17,21,23 and 26) go to Node 2. This indicates the point discussed in variable importance results, that the variable of "cause of crash" is the best factor to classify the injury severity in traffic crashes which take place on freeways. At both nodes 1 and 2 the next splitter is seatbelt use; sending the data related to using seat belt to the left side (Node 3 and 5), and the data related to not using seat belt or unknown condition of usage to the right (Node 4 and 6). The left branch of the tree shows that while a crash takes place due to causes No. 1,2,3,11,13,14,15,16,19,20 and 22, if a driver uses a seat belt, there is a higher probability he will be safe (terminal node 3); otherwise, he may get injured (terminal node 4).

Turning to the left branch of the tree, node 5 is divided into terminal nodes 7 and 8, based on the "collision type" variable. As indicated by terminal node 8, when collision type is fire/explosion or overturning (cases No. 7 and 8), the likelihood of being injured is the highest, even if the driver is using a seat belt. This may be due to lack of paying serious attention to the safety of roadsides, which are in poor condition. The classification tree then splits node 6 again by the variable of "cause of crash", directs the cases of 5,6,7,23 and 26 to the right, forming terminal node 10; while directs the cases 4,17 and 21 to the left, forming terminal node 9. The tree analysis has classified node 9 under fatality class and node 10 under injury class. This, at first glance, indicates the importance of using a seat belt, since both nodes have initiated from node 6, where the drivers have not used a seat belt. It shows that not using a seat belt significantly increases the probability of injury or death, and results in more severe crashes. Moreover, focusing on terminal node 9, causes of No. 4, 17 and 21 corresponding Inattention to traffic ahead, Vehicle defect, and movement of pedestrians, livestock and unauthorized vehicles on freeways are revealed as fatal causes of crash in freeways. Inattention to traffic ahead (cause No. 4) is also one of the serious causes of increasing injury severity on freeways, because on such roads people are usually driving at very high speeds. For instance, a vehicle with the speed of 140 km/h moves about 40 meters a second, and therefore one second of neglect can cause a disaster.

## Conclusion 

The study indicated that "seat belt use", "cause of crash" and "collision type" are the most important factors influencing the injury severity of drivers on rural roads.

This study indicates that in Iran, which has a high rate of traffic crash fatality, due to a poorly developed driving culture, seatbelt use is still the most important factor in increasing injury severity. But on freeways, where there is strict police enforcement and control of seat belt use, the importance of this variable is less.

Cause of crash, also turned out to be the other important variable. To be more precise, the analysis revealed that for two-lane, two-way rural roads, improper overtaking and speeding are the most serious causes of increasing injury severity, and since overtaking takes place by using the opposite lane, it results in more severe injuries and more lives lost. Moreover, "inattention to traffic ahead", "vehicle defect", and "movement of pedestrians, livestock and unauthorized vehicles on freeways", were found as the most serious causes of crashes which result in higher injury severity among drivers on freeways. To deal with these problems, intensifying the police enforcement by means of mobile patrol vehicles, constructing overtaking lanes where required, and prohibiting the crossing of pedestrians and livestock and of the presence of unauthorized vehicles on freeways are necessary.

On the other hand, "fire/explosion" and "overturning" are found to be the most dangerous collision types. In order to prevent such events and improve the overall safety of freeways, paying serious attention to the safety of roadsides is recommended. One remedial measure that can be applied in this regard is creating a rumble strip on the two edges of roads, which is very rarely done in the freeways at the present time. Furthermore, most of Iran’s freeways are so straight and long that they make the drivers tired and sleepy, which has dangerous consequences. Paying attention to the design consistency of such roads can be a helpful issue in solving this problem.
